# Rethinking the Applications of Ionic Liquids and Deep Eutectic Solvents in Innovative Nano-Sorbents

**DOI:** 10.3389/fchem.2021.653238

**Published:** 2021-04-09

**Authors:** Lirong Nie, Sara Toufouki, Shun Yao, Dong Guo

**Affiliations:** ^1^School of Medical Instrument and Food Engineering, University of Shanghai for Science and Technology, Shanghai, China; ^2^Department of Pharmaceutical and Biological Engineering, School of Chemical Engineering, Sichuan University, Chengdu, China

**Keywords:** ionic liquids, deep eutectic solvents, nano-sorbents, separation, extraction

## Abstract

With the development of green chemistry and nano materials, new alternatives to traditional volatile solvents are one of many important hotspots in the field of nano materials. Ionic liquids (ILs) and deep eutectic solvents (DESs) as excellent alternative solvents are being applied in the innovation of nano-sorbents, including nanoparticles, nanogels, and nanofluid. ILs and DESs are often used as carriers/modifiers/dispersers of nano-sorbents to enhance the adsorption capacity and selectivity in the extraction procedure. Various extraction technologies, such as solid-phase extraction, solid-phase microextraction, micro-solid phase extraction, hollow fiber liquid phase microextraction, and magnetic solid-phase extraction, have also been promoted by them to achieve rapid development. This paper focused on the latest development of nano-sorbents based on ILs and DESs. The problems and bottlenecks encountered were analyzed in order to provide meaningful and valuable references for the related research and thus promote further development and application of alternative solvents-assisted nano-sorbents.

New alternative solvents have gained increasing attention in research due to the negative impact of traditional chemicals on people and the environment. Therefore, they are employed as eco-friendly extractants and sorbents to replace the old ones (Sepulveda et al., 2020). Mainly, ionic liquids (ILs) and deep eutectic solvents (DESs) contribute greatly to separation science besides their use as an extraction medium (Marták and Schlosser, 2019); they can also assist adsorbents to improve separation performance by the disaggregation of nano-materials into smaller bundles (Zaib et al., 2020).

Generally, DESs are composed of Brønsted or Lewis acids and bases. They are recognized as “greener alternatives” to conventional solvents due to their excellent properties, as they are biodegradable, non-toxic, and safe. Among them, the DES choline chloride-glycerol (ChCl-Gly) was reported to be truly “green” due to the absence of metal salts in their composition. It has been proved that aqueous ChCl-Gly is among the most readily biodegradable and is “practically harmless” to the aquatic ecosystem (Juneidi et al., 2015). Additionally, newer generations, i.e., cholinium-derived ILs were chosen as the component of an aqueous two-phase (ATP) to simultaneously extract and recover antioxidants and carbohydrates from food waste (Neves et al., 2019). These used ILs were proven to have low toxicity toward human intestinal epithelial cells (Caco-2 cell line).

However, the components of ATP extraction are limited and emulsion easily occurs, meanwhile the appropriate conditions for phase separation are usually inconsistent with those for extraction. Besides, the targets often exist in the high boiling-point phase with sugars or salts, which is hard to recover. Finally, the composition of the two phases will be changed greatly after extraction, which is more appropriate for one-time use because of hard recycling.

Immobilization of alternative solvents on sorbents is an effective solution for their residue, difficult recovery, and high consumption problems; it also avoids the mentioned weakness of ATP extraction. Consequently, a series of innovative nano-sorbents are being invented. Recently, some articles in Frontiers in Chemistry have documented the combination of the above alternative solvents with the zeolitic imidazolate framework (ZIF-8, Liu et al., 2019; Ferreira et al., 2020), graphene (Edvaldo et al., 2020), and so on. These studies reinforce the use of ILs or DESs in nanoscale for sample preparation with magnetic solid-phase extraction (MSPE), solid-phase microextraction (SPME), and micro-solid phase extraction (MSPE). More specifically, IL-graphene-based materials (ILGBMs) show excellent extraction efficiency of analytes in numerous matrices compared to the basic form. Furthermore, scientists utilized reduced graphene oxide (rGO)-assisted ILs-based nanofluid to extract trace estrogens in milk samples (Chu et al., 2019). Another study proved that silica-coated magnetic cobalt nanoparticles covered with an external layer of [P_66614_][BEHPA] are an excellent sorbent to extract Pb from the water (Hosseinzadegan et al., 2019). A ZIF-8-based composite modified by diethyl phthalate exhibited high extraction capacity for MSPE of pyrethroid pesticides in environmental water (Liu et al., 2019).

Some problems were reported when using graphene as sorbents in solid-phase extraction (SPE), such as irreversible aggregation and long equilibrium time, which may decrease the adsorption capacity of the sorbent. To overcome these problems, innovative nano-sorbents that combined the multiple active sites of ILs and the magnetic separation of magnetic nanoparticles (MNPs) were developed. It was recently reported that a bucky nanogel was prepared by the simple mixture of ILs with magnetic graphene oxide (MGO) (Jamshidi et al., 2020). This nanogel can be used as a nano-solvent to remove heavy and hazardous metal ions from wastewater, showing excellent absorption capacity. In this study, the IL of [BMim][PF_6_] was bonded to the surface of the carbon nanoparticles by strong π-π interactions. Additionally, the IL [P_4444_][2-Op] as modifiers was loaded into the mesoporous silica vesicle (MSV) with different pore structures for fabricating nano-sorbents (ionogels, Xue et al., 2019). In this case, the adsorption performances of nano-sorbents mainly ascribed to the loading level and the dispersing state of ILs. Pore diameter as well as pore volume of the supports directly determined the adsorption capacity and rate. Although the support with a small mesopore benefitted when forming monomolecular layers for the lowly loaded IL due to their strong nano-confinement effect, the nano-sorbents formed were easily blocked by highly loaded IL. In practical application, ILs should be dispersed as a monomolecular layer if possible on the supports to expose more active sites, which is beneficial for achieving fast and efficient adsorption.

Polymerized ILs (PILs) are also very popular in the application of innovative nano-sorbents. Nevertheless, most polymerized ILs (PILs) contain only one kind of functional group in the cationic part. In order to increase the adsorption sites, a new PIL with other bonded functional groups in the cationic part was reported and used to prepare a PIL-functionalized magnetic mesoporous silica nano-sorbent (PILs@mSiO_2_@nSiO_2_@Fe_3_O_4_, Zhao et al., 2019). The magnetite core makes the nano-sorbent easy to separate, and the mesoporous silica endows the material with a high pore volume and large surface area. Moreover, the bonded functional groups can perfectly match the chemical structure of the analytes to improve adsorption efficiency. In addition, cyclodextrin (CD) with fascinating properties, such as a hydrophobic inner cavity and a hydrophilic exterior, has successfully attracted the attention of researchers. The research group of Raoov reported poly(cyclodextrin-ionic liquid)-based magnetic nanoparticles as nano-sorbents to extract polycyclic aromatic hydrocarbons from food samples (Boon et al., 2019, 2020). Besides the high extraction capacity, the magnetic nano-sorbent could be reused several times with its performance remaining practically constant.

Multi-wall carbon nanotubes (MWCNTs) have great potential applications in adsorption materials due to their porous and hollow structure, large specific surface area, high thermal stability, and easy surface modification. However, MWCNTs are easily agglomerated in solution and difficult to disperse in the matrix, which reduces the specific surface area and the adsorption capacity. The above problems can be solved effectively by immobilization of green hydrophobic ILs on the magnetic MWCNTs (Tarigh et al., 2020). This green reusable nano-sorbent was employed successfully for the extraction of chromium species. For the selection of ILs, it mainly depends on the nature of MWCNTs and target analytes. The research of MWCNTs modified by ILs is an emerging field, which is still in its infancy. The potential of ILs in the field of nanomaterials has not yet been fully exploited. The cost and sustainability issues in practical applications have restrained its rapid development. With the continuous innovation of the chemical industry and ILs, more and more alternative solvents-modified nano-sorbents will be synthesized.

In hollow fiber liquid phase microextraction (HF-LPME), the mass transfer process of the target analyte is mainly dependent on passive diffusion and its main drawback is the long extraction time. Introduction of an electric field into the above extraction procedure is an effective method for reducing the extraction time and eliminating the interference of the matrix. As a new alternative micro scale technique, electro membrane extraction (EME) has been applied in the pretreatment of biological samples since it was proposed in 2006. In this method, combination of ILs on an MWCNTs/ZnO nano-hybrid as a nano-sorbent is a new, effective strategy for extraction and quantification of imatinib mesylate (IMM) in human plasma (Forough et al., 2017). The applied nanocomposite ([OMIm]Br-MWCNTs@ZnO)-reinforced hollow fiber exhibited excellent extraction performance. This is largely attributed to the fact that the used IL is a heterocyclic compound containing nitrogen with electron-rich group, which could interact with target analytes via numerous interactions, such as hydrogen bonds, π-π conjugation, anion exchange, hydrophobic interactions, and so on. Although IL-supported nanocomposites have been successfully used as nano-sorbents, there is no scientific basis for the selection of ILs at present, and most of them are based on experiments. Researchers can select specific ILs according to the properties of the target analyte. In addition, the electrical conductivity of ILs may also affect the extraction efficiency of nano-sorbents modified by ILs in the EME method. Therefore, it is still one of the more important hot topics to search for ILs with higher conductivity for the preparation/modification of nano-sorbents.

Besides ILs, many DESs can also be used as carriers/modifiers/dispersers of nanoparticles to enhance the affinity and selectivity between the nano-sorbents and the analytes in the extraction process. Conventional nanoparticles as sorbents have many limitations in practical applications due to their shortcomings, such as single performance and difficulty in separation and recovery. Recently, some literature have confirmed that the combination of DESs and magnetic nanoparticles (MNPs), such as Fe_3_O_4_-DES (Liu et al., 2020) and SiO_2_@Fe_3_O_4_-DES (Majidi and Hadjmohammadi, 2019), have a broad application prospect in the magnetic solid-phase extraction method, owing to the advantages of their small particle size, large specific surface area, reproducibility, environmentally friendly materials, and easy solid-liquid separation. Compared with traditional nano-sorbents, magnetic nano-sorbents can effectively improve extraction efficiency. Therefore, exploitation of innovative magnetic nano-sorbents modified by alternative solvents will be the focus of research in the future. At present, most of the research on MNPs containing alternative solvents are limited to the laboratory. Further comprehensive evaluation of their feasibility and economic benefits is needed for large-scale industrial applications. Moreover, many studies have pointed out that magnetic nano-sorbents are regenerative and can be reused (Shahriman et al., 2018; Lu et al., 2020; Dil et al., 2021). However, few studies have carried out in-depth characterization of regenerated nano-sorbents and provided corresponding optimization and adjustment schemes to increase the service life of the nano-sorbents or maintain a certain adsorption performance (Alosmanov et al., 2018). As a summary, recent applications and preparation of innovative nano-sorbents based on alternative solvents are shown in Table 1 and Figure 1, respectively.

**Table 1:** Recent applications (2019–2020) of alternative solvents in innovative nano-sorbents.

**Sorbent**	**Alternative solvent**	**Extraction technique**	**Analysis technique**	**References**
Magnetic multi-wall carbon nanotube (MMWCNT-IL)	[A336][TS]	DMSPE	UV-Vis	Tarigh et al., 2020
Magnetic grapheneoxide nanogels	[BMim][PF_6_]	DSPE	CV-AAS	Jamshidi et al., 2020
GO-assisted IL-based nanofluid	[C_4_MIM][PF_6_], [C_6_MIM][PF_6_], [C_8_MIM][PF_6_]	DLLME	HPLC	Chu et al., 2019
Magnetic nanoparticles coated with IL	[P_66614_][BEHPA]	SPE	FI-ICP-OES	Hosseinzadegan et al., 2019
Ionic liquid magnetic graphene (IL@MG)	[C_4_MIM][Br], [C_4_MIM][BF_4_]	MSPE	UHPLC-MS/MS	Liu et al., 2019
Magnetic mesoporous nanoparticles (PILs@mSiO_2_@nSiO_2_@Fe_3_O_4_)	Cyano and phenyl groups-based PILs	MSPE	GC/MS	Zhao et al., 2019
Magnetic ferrofluid	Poly(βCD-IL)	DLPM	GC-FID	Boon et al., 2020
Magnetic poly(β-CD-IL)nanocomposites(Fe_3_O_4_@βCD-Vinyl-TDI)	β-CD-Vinyl-OTs	MSPE	GC-FID	Boon et al., 2019
Nano-Fe_3_O_4_ particles (Fe_3_O_4_-DES)	Urea, ethylene, glycol, oxalic acid, glycerol	MSPE	ICP-OES	Liu et al., 2020
Magnetic nanoparticles (SiO_2_@Fe_3_O_4_-DES)	Ethylene glycol, tetramethylammoniumchloride	M-D-μSPE	HPLC	Majidi and Hadjmohammadi, 2019
Magnetic zeolitic imidazolate framework-8@DES (M-ZIF-8@DES)	1-Methy-3-octyl imidazolium chloride, 1-undecanol	MSPE	GC-MS/MS	Liu et al., 2019
Single walled carbon nanotubes (SWNTs)	Choline chloride, glycerol	–	–	Zaib et al., 2020

**Figure 1:**
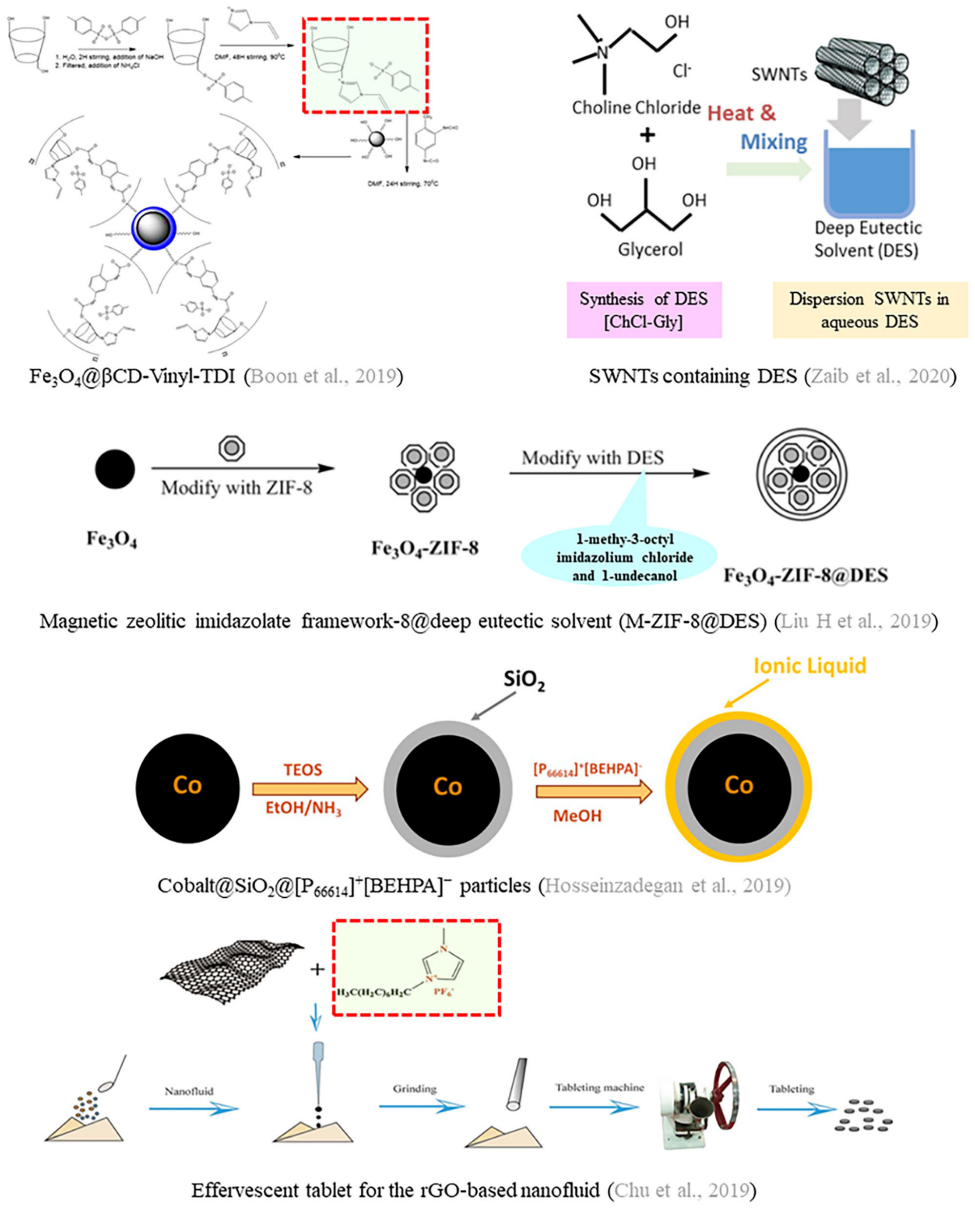
Preparation of typical nano-sorbents based on green solvents.

As a whole, all of the above supports the fact that alternative solvents can be effectively applied in various innovative nano-sorbents or to improve the existing ones. The form of sorbents can be expanded to membranes, microcapsules, nanogels, and so on. Besides nanoparticles, both the target recognition and adsorption capacity need further improvement. The combination of alternative solvents on sorbents can be more flexible and simpler; broader comparison together with evaluation for their performance are expected. It is assumed that a series of commercial IL/DES-based nano-sorbents will come out soon.

The following are suggestions for rethinking before their further application: (1) they should be coupled with green reagents to ensure that the whole process is green; (2) they should be used as recyclable resources to reduce their high cost, which makes them more practically applied in microextraction and adsorption for analytical purposes; (3) although the synthesis, modification, and application of nano-sorbents based on ILs and DESs have made some achievements, further theory research and practiced directions are needed to target the development of new, green, and sustainable nano-sorbents; (4) from the perspective of nano-sorbents preparation, most of the preparation methods are chemical methods, which may cause pollution in the application process. Therefore, cellulose, clay, starch, and other environment-friendly materials should be considered to combine with ILs or DESs to prepare nano-sorbents; and (5) in terms of technology, there is a lack of a desorption method to recover used nano-sorbents. If this technology can be well-developed, the economic benefits of nano-sorbents will be doubled. Unfortunately, the research in this area is very limited at present. It is necessary to explore an effective method to restore the activity of nano-sorbents, and the “resurrected” nano-sorbents should have a large specific surface area to ensure their adsorption efficiency.

## Data Availability Statement

The original contributions presented in the study are included in the article/Supplementary Material, further inquiries can be directed to the corresponding author/s.

## Author Contributions

All authors listed have made a substantial, direct and intellectual contribution to the work, and approved it for publication.

## Conflict of Interest

The authors declare that the research was conducted in the absence of any commercial or financial relationships that could be construed as a potential conflict of interest.
